# Hyaluronated and PEGylated Liposomes as a Potential Drug-Delivery Strategy to Specifically Target Liver Cancer and Inflammatory Cells

**DOI:** 10.3390/molecules27031062

**Published:** 2022-02-04

**Authors:** Stefania Cannito, Valeria Bincoletto, Cristian Turato, Patrizia Pontisso, Maria Teresa Scupoli, Giorgia Ailuno, Ilaria Andreana, Barbara Stella, Silvia Arpicco, Claudia Bocca

**Affiliations:** 1 Department of Clinical and Biological Sciences, University of Turin, 10125 Turin, Italy; stefania.cannito@unito.it (S.C.); claudia.bocca@unito.it (C.B.); 2 Department of Drug Science and Technology, University of Turin, 10125 Turin, Italy; valeria.bincoletto@unito.it (V.B.); ilaria.andreana@unito.it (I.A.); barbara.stella@unito.it (B.S.); 3 Department of Molecular Medicine, University of Pavia, 27100 Pavia, Italy; cristian.turato@unipv.it; 4 Department of Medicine, University of Padova, 35128 Padova, Italy; patrizia@unipd.it; 5 Research Center LURM, Interdepartmental Laboratory of Medical Research, University of Verona, 37134 Verona, Italy; mariateresa.scupoli@univr.it; 6 Department of Neurosciences, Biomedicine and Movement Sciences, University of Verona, 37129 Verona, Italy; 7 Department of Pharmacy, University of Genova, 16148 Genova, Italy; ailuno@difar.unige.it

**Keywords:** HCC, CD44, hyaluronic acid, liposomes, macrophages

## Abstract

Hepatocellular carcinoma (HCC) is the most frequent primary liver cancer and is characterized by poor clinical outcomes, with the majority of patients not being eligible for curative therapy and treatments only being applicable for early-stage tumors. CD44 is a receptor for hyaluronic acid (HA) and is involved in HCC progression. The aim of this work is to propose HA- and PEGylated-liposomes as promising approaches for the treatment of HCC. It has been found, in this work, that CD44 transcripts are up-regulated in HCC patients, as well as in a murine model of NAFLD/NASH-related hepatocarcinogenesis. Cell culture experiments indicate that HA-liposomes are more rapidly and significantly internalized by Huh7 cells that over-express CD44, compared with HepG2 cells that express low levels of the receptor, in which the uptake seems due to endocytic events. By contrast, human and murine macrophage cell lines (THP-1, RAW264.7) show improved and rapid uptake of PEG-modified liposomes without the involvement of the CD44. Moreover, the internalization of PEG-modified liposomes seems to induce polarization of THP1 towards the M1 phenotype. In conclusion, data reported in this study indicate that this strategy can be proposed as an alternative for drug delivery and one that dually and specifically targets liver cancer cells and infiltrating tumor macrophages in order to counteract two crucial aspect of HCC progression.

## 1. Introduction

Hepatocellular carcinoma (HCC) is the most frequent primary liver cancer. In particular, it is ranked as the fifth most common malignancy and the fourth leading cause of cancer-related mortality worldwide, with an incidence that continues to increase [1,2]. HCC, whatever the etiology, usually develops in conditions of chronic liver disease (CLD), mostly in the context of a cirrhotic liver, where inflammation and fibrosis promote tumor progression and resistance to therapy [3]. Although cirrhosis is still the main risk factor for HCC development, alarming recent data also indicate increasing numbers of HCC cases being diagnosed in earlier stages, non-cirrhotic, of non-alcoholic fatty liver disease (NAFLD) patients. NAFLD is currently the most frequent CLD in industrialized countries (prevalence: approx. 20% among the general population, >70% among obese and/or diabetic type II and/or metabolic syndrome patients) and is closely associated with insulin resistance and oxidative stress. Between 20% and 30% of NAFLD patients develop non-alcoholic steatohepatitis (NASH), which is accompanied by a chronic inflammatory response and fibrogenesis that finally lead to liver injury and eventually progress to HCC [4,5,6]. Although the molecular mechanisms of NAFLD progression are largely unknown, it is currently thought that NAFLD is a multistep disease, in which multiple parallel pathogenic events act synergistically, and not as a consequence of each other, to promote NASH evolution and eventually HCC development. Any single event/player that contributes to NAFLD/NASH-related HCC development, including inflammation (i.e., inflammatory cells) and tumor/stroma interactions, can thus be considered a potential therapeutic target.

Emerging evidence has shown that the surrounding tumor microenvironment (TME), which is known to exert a notable influence on HCC development, should also be considered in the management of the disease. In this scenario, the inflammatory microenvironment and, significantly, tumor-associated macrophages (TAMs) should be considered as main factors able to influence HCC progression [7]. In particular, TAMs are able to change their functional profile and phenotype in response to environmental signals, with there being two main types of macrophages, termed M1 and M2 [8]. M2 macrophages are usually believed to promote tumorigenesis and tumor progression, while M1 macrophages are thought to be pro-inflammatory and tumoricidal [9]. However, there are increasing amounts of data to demonstrate that M1 macrophages may also have pro-tumor functions. For instance, M1 macrophages induce the epithelial–mesenchymal transition of pancreatic ductal adenocarcinoma cells [7], and enhance the motility of HCC cells [10]. 

Polymeric nanoparticles, liposomes, and micelles carrying small molecules, proteins, peptides, and nucleic acids have attracted great attention for the treatment of various cancers including HCC. Herein, we discuss the pathogenesis of HCC in relation to its various recent treatment methodologies using nanodelivery of monoclonal antibodies (mAbs), small molecules, miRNAs, and peptides.

In this complex scenario, Dutta and Mahato [11] have provided a synopsis of selected recent treatment methodologies using nanomedicines [12], including liposomes, micelles, and polymeric nanoparticles, that carry drugs as a new strategy and a promising approach for the successful treatment of HCC in early stage patients. Nanomedicine is a recent development that is used to improve the antitumor effect of chemotherapeutics on cancer cells by delivering elevated drug concentrations to tumor tissue and improving the pharmacokinetic and pharmacodynamic characteristics of those drugs. Liposomes have undergone widespread investigations as they are biocompatible and biodegradable, mimic cell-membrane properties, and are able to deliver compounds with different physico-chemical characteristics [13]. Different preparation methods have been described and a recent approach deals with a one-step process to produce liposomes using supercritical carbon dioxide [14]. Several liposomal formulations are currently on the market or in clinical trials [15,16,17]. Moreover, several studies have dealt with the modification of liposome surfaces in order to improve the targeting ability of the systems towards specific cells or tissues [18,19].

With regards to the liver, the presence of highly permeable vasculature in HCC together with the possibility of directly targeting cancer cells using decorated vesicles with ligands for HCC-specific antigens or receptors is potentially a winning strategy for simultaneously acting against tumor cells and preventing damage to healthy tissue [20]. On the other hand, nanoparticles are also able to both induce and inhibit immune-system components [21]. In particular, liposome–macrophage interactions have been extensively studied [21] with an eye to two contrasting therapeutic goals: (i) the design of nanoparticles that cannot be detected by phagocytic cells, as macrophages can recognize nanoparticles and remove them from plasma, thus reducing their ability to act as effective drug carriers; (ii) the internalization of nanoparticles by macrophages can be considered a drug-delivery strategy, as they can migrate in tissue while carrying therapeutic drugs. In addition, a nano-formulated delivery system with the ability to influence macrophage polarization has good potential for providing benefits in a variety of disease states. 

A large amount of research [11] has been carried out on various receptors to improve drug-delivery carrier systems, and these include the following: (i) lactosylated liposomes that encapsulate calcein/doxorubicin; (ii) RGD (arginine-glycine-aspartate)-coupled liposomes to target integrin receptors in HCC; (iii) liposomes coated with folic acid to improve targeting ability towards the folate receptor; and (iv) glycyrrhetinic acid (GA)-modified liposomes that are a potential target for drug delivery in HCC as they exploit GA-receptor overexpression on the hepatocyte surface [22]. 

Along these lines, the cluster of differentiation 44 (CD44) antigen is a cell-surface glycoprotein receptor that can modulate several biological functions, including hematopoiesis, lymphocyte activation, recirculation, and homing, as well as tumor progression and metastasis. This transmembrane glycoprotein is a receptor for hyaluronic acid (HA), but also binds osteopontin, collagen, and fibronectin, as well as acting as a co-receptor for epidermal growth factor receptor (EGFR) and c-Met [22,23]. In addition, through its interaction with HA, CD44 is involved in monocyte differentiation as well as in several macrophage-mediated functions, such as adhesion to extracellular matrix, phagocytosis, chemotaxis to inflammatory sites, and the secretion of cytokines [24].

CD44 has been introduced as a cancer stem cells marker in HCC together with CD90, CD133, CD24, and EpCAM, and is reported to be involved in tumor initiation and growth, cancer progression, and promoting metastasis [22,23]. Moreover, literature meta-analysis data show that CD44 is associated with tumor stage (evaluated with the Classification of Malignant Tumors, TNM) and has been suggested to be an independent factor in reduced overall survival, with its expression correlating with worse prognosis in HCC patients [22,23]. 

The targeting of liver cancer cells and inflammatory cells that overexpress the CD44 receptor using modified liposomes may thus be a strategy that can emphasize drug delivery and minimize toxic effects to healthy tissues. While bearing in mind the complex scenario of HCC, this study aims to analyze the effects of hyaluronated and PEGylated liposomes in liver cancer cells as well as in human and murine macrophage cell lines with the aim of proposing a more efficient therapeutic treatment.

## 2. Results

### 2.1. Liposome Preparation and Characterization 

Small unilamellar HA-decorated liposomes were prepared by the addition of the HA 4800-DPPE (HA 4800) or HA 17000-DPPE conjugates (HA 17000), in different batches, at molar ratios of 3 and 5, during the hydration phase of the lipid films, which were either composed of 1,2-distearoyl-sn-glycero-3-phosphocoline (DSPC), cholesterol (CHOL), and 1,2-distearoyl-sn-glycero-3-phosphoethanolamine-*N*-[amino(polyethylene glycol)-2000] (mPEG2000-DSPE, PEG) (55:40:2 molar ratio) or only DSPC/CHOL (55:40 molar ratio). With this preparation method, the phospholipid chain was incorporated into the liposome membrane, while the HA was exposed toward the aqueous phase. Plain liposomes were composed of DSPC, CHOL, and either PEG or l-α phosphatidyl-dl-glycerol sodium salt (PG, indicated as ND in the text) in a 55:40:5 molar ratio.

The physico-chemical characteristics of the liposomes are reported in Table 1. Liposomes displayed a dimensional range of about 165 nm to 225 nm with low PDI (<0.2), and the particle size of the HA-liposomes tended to increase with the addition of the HA-DPPE conjugate and with increasing polymer molecular weight, as has already been reported [25]. The zeta potential was negative for all formulations; at around −15 mV for uncoated liposomes (PEG and ND) and at more negative values for HA-decorated analogues owing to the presence of the negative carboxylic residues of HA on their surface. Specifically, the values ranged from about −20 mV to −40 mV depending on the amount of HA-DPPE conjugate in the formulation and on the molecular weight of HA (Table 1). 

### 2.2. Expression of CD44 in Human HCC and Experimental Model of Hepatocarcinogenesis

**Figure 1:**
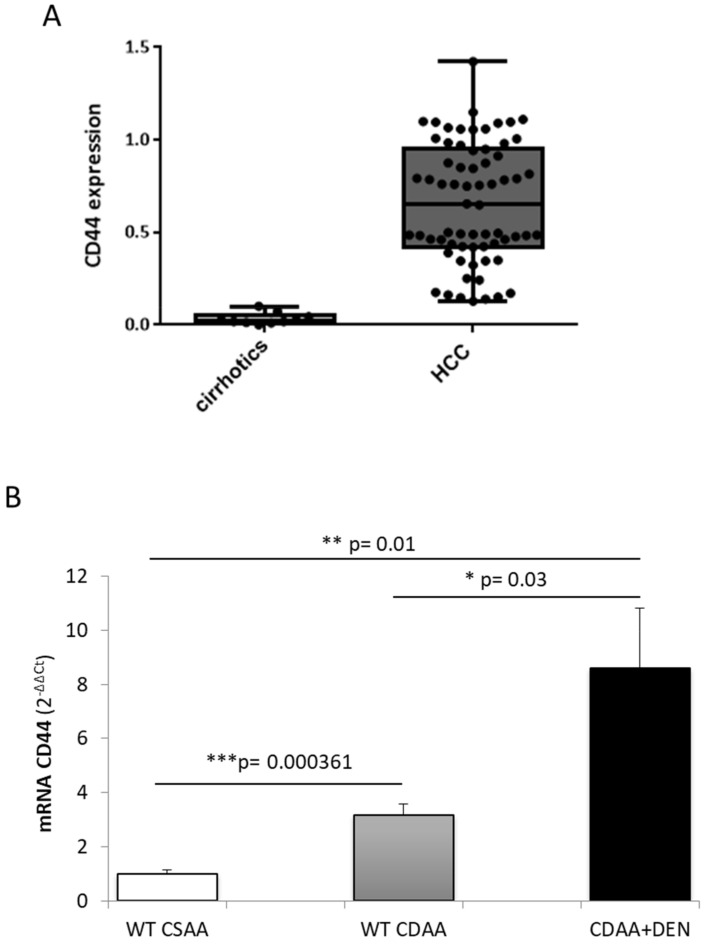
(**A**,**B**) In-vivo expression of the CD44 receptor. (**A**) Quantitative real-time PCR (qPCR) analysis of CD44 transcript levels in cirrhotic patients vs. HCC patients of mixed etiology. Mann–Whitney test of frequency distribution data. Boxes include the values within the 25th and 75th percentiles, whereas the horizontal bars represent the medians. The extremities of the vertical bars indicate the minimum and the maximum value. (**B**) qPCR analysis of CD44 transcripts in wild type C57BL/6 submitted to the DEN/CDAA carcinogenic protocol or fed the CDAA diet in comparison with corresponding littermates fed the CSAA control diet. Statistical analysis was performed using the Kruskal–Wallis test of one-way ANOVA data with Dunn’s correction for multiple comparisons of frequency distribution data (* *p* < 0.05, ** *p* < 0.01, and *** *p* < 0.001).

According to the aim of this study, we first analyzed CD44 expression in a cohort of human patients with cirrhosis (*n* = 10) and in HCC patients of mixed etiology (*n* = 67), including HCC related to metabolic syndrome diseases (NAFLD/NASH). As shown in Figure 1A, CD44 transcript levels are significantly higher in HCC patients than in those with cirrhosis. Moreover, in order to further investigate the relevance of CD44 in NAFLD-related HCCs, we evaluated the expression of the CD44 receptor in mice with experimental NASH, which was induced by feeding animals a choline-deficient L-amino acid-defined (CDAA) diet, as well as in mice carrying hepatocarcinoma that originated in a NASH background under the DEN/CDAA carcinogenic protocol. First of all, we observed that CD44 transcript levels are higher in the livers of animals with NASH (fed a CDAA diet) than in the control mice and, more significantly, that they are up-regulated in DEN/CDAA-induced tumors compared with either control mice and experimental NASH (Figure 1B).

### 2.3. CD44 Expression in Human Hepatic Cancer Cell Lines

As the in vivo results demonstrated that CD44 is overexpressed in hepatocellular carcinoma, the next step was to evaluate the expression of this receptor in human liver cancer cell lines. In this connection, we analyzed two different cancer cell lines, HepG2 and Huh7, which potentially mimic in vitro two different grades of liver tumor, with HepG2 as the hepatoblastoma cell line model, well differentiated and with low deviation, and Huh7 as hepatocarcinoma cells, which are a more aggressive and invasive liver cancer cell line.

**Figure 2:**
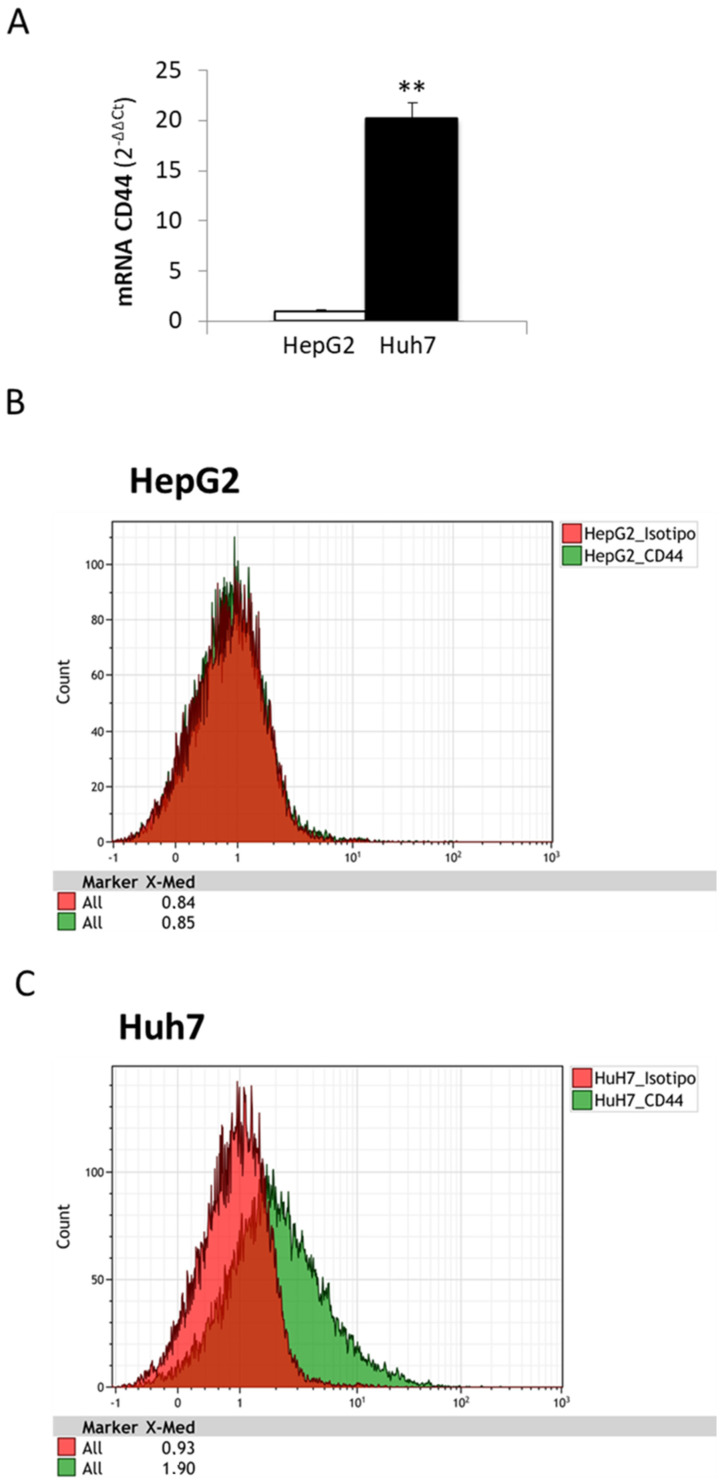
(**A**–**C**) In vitro expression of CD44 in liver cancer cell lines. (**A**). Comparison of CD44 mRNA levels in HepG2 and Huh7 using qPCR. Data in graphs are expressed as means ± SD (** *p* < 0.01). (**B**,**C**) Flow cytometry analysis of CD44 expression on plasma membranes of HepG2 (**B**) and Huh7 (**C**). Isotype-matched antibody (isotype) was used as the control.

As shown in Figure 2A, the CD44 transcript is expressed at very low levels in HepG2 cells (almost close to zero), while we observed a consistent (up to 20-fold increase vs. HepG2 cells) and significant increase in CD44 mRNA in Huh7 cells. This difference in CD44 expression levels was also confirmed at the protein level by analyzing the expression of this receptor on the plasma membranes of liver cancer cells. Flow cytometry analyses performed on HepG2 and Huh7 revealed that CD44 expression on HepG2 cells is overlapped to the isotype-matched control antibody, whereas Huh7 cells showed an evident shift in the histogram, confirming the more abundant expression of CD44 on its surface (Figure 2B,C). These results indicate that CD44 expression on membrane surfaces increases in parallel with the aggressiveness of tumor cells, and confirm that both HepG2 and Huh7 cells are suitable in vitro models to study the relevance of this receptor in liposome uptake. 

### 2.4. Uptake of Hyaluronic Acid-Modified Liposomes in Human Hepatic Cancer Cell Lines

**Figure 3:**
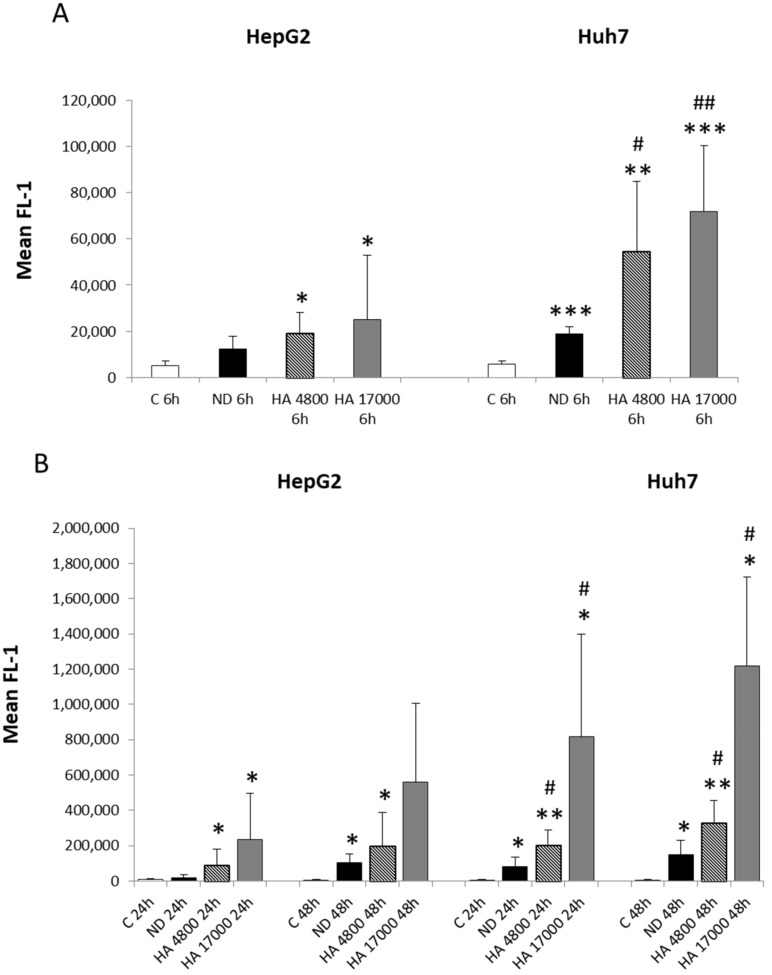
(**A**,**B**) Cellular uptake of different formulations of HA-decorated fluorescein-5-isothiocyanate (FITC)-labeled liposomes in liver cancer cells. Time-course flow cytometry analysis of internalization of fluorescent liposomes in either HepG2 and Huh7 at early time (6 h, panel **A**) and at later times (24–48 h, panel **B**), after incubation of cells with either non-decorated liposomes (ND) or synthesized HA-liposomes with two different molecular weights (HA 4800 or HA 17000). Data in graphs are expressed as means ± S.D of three independent experiments (* *p* < 0.05, ** *p* < 0.01, *** *p* < 0.001 vs. control; ^#^ *p* < 0.05, ^##^ *p* < 0.001 vs. ND liposomes).

As previous studies have reported the use of modified liposomes as a satisfactory and efficient technique for delivering drugs to liver cancer cells as they can elude the delivery restrictions implemented by the reticuloendothelial system (RES) after systemic administration [22], we decided to evaluate the effect of HA, which is ligand of the CD44 receptor, on the cellular uptake of liposomes. For this purpose, we prepared the following liposome formulations: (a) non-decorated liposomes (ND); (b) liposomes decorated with HA at a molecular weight of 4800 (HA 4800); and (c) liposomes decorated with HA at a molecular weight of 17000 (HA 17000). We then investigated the cellular uptake of these different liposome formulations using flow cytometry. Our results show that HepG2 and Huh7 are able to uptake liposomes in a time-dependent manner (Figure 3A,B) as compared with control cells. Specifically, HepG2 cells show a significant increase in the internalization of hyaluronated liposomes from 6 to 48 h, compared with the HepG2 control cells, with values of mean FL-1 ranging between 12,000 and 560,000. However, no significant differences are observed between hyaluronated and non-decorated (ND) liposomes’ uptake at any of the analyzed time points (Figure 3A,B). By contrast, we observe significant differences in the internalization of hyaluronated liposomes by Huh7 cells, compared with non-decorated liposomes, starting from as early as 6 h (Figure 3A) and continuing up to 48 h (Figure 3B), with values of mean FL-1 ranging from 54,000 to 1,200,000. Of relevance, the uptake of HA-modified liposomes in Huh7 is significantly higher than in HepG2 cells, suggesting that liposomes internalization occurs through different mechanisms in the liver cancer cell lines taken into consideration.

**Figure 4:**
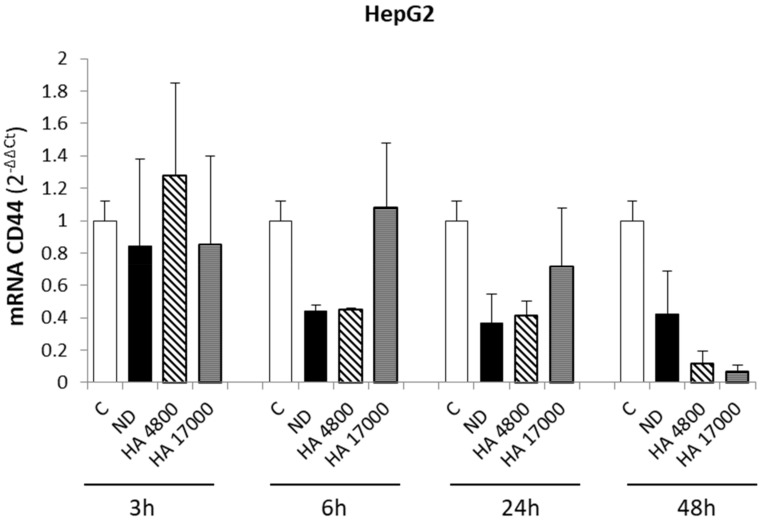
Modulation of CD44 expression on HepG2 cells after treatment with synthesized liposomes. Quantitative real-time PCR (qPCR) analysis of CD44 expression in HepG2 cell line after incubation with liposome formulations at the indicated time points. ND = non-decorated liposomes, HA 4800 and HA 17000 = synthesized HA-liposomes with two different molecular weights. Data in graphs are expressed as means ± S.D of three independent experiments. No significant differences were observed.

These data are consistent with the CD44 transcript levels, as analyzed by qPCR, which revealed no differences in CD44 expression in HepG2 cells after treatment with HA-decorated liposomes (Figure 4). These results suggest that the entry of liposomes into HepG2 cells is due to passive diffusion through the plasma membrane, which is consistent with the almost total absence of the CD44 receptor on the HepG2 surface. On the other hand, the significant increase in the uptake of HA-decorated liposomes in Huh7 demonstrates that CD44 can improve liposome uptake in aggressive liver cancer cells, making it a specific and suitable receptor for targeted drug delivery with liposomes that can distinguish between liver cancer cell lines and healthy hepatic cells. 

### 2.5. Uptake of Macrophage Cell Lines

To evaluate the therapeutic efficacy of liposomes, it is important to consider two distinct critical aspects: (i) cellular uptake by target cells; (ii) interaction with immune system, in which macrophages play an important role, especially for drug delivery.

To this aim, we synthesized modified liposomes that were coupled with (i) polyethylene glycol (PEG); (ii) hyaluronic acid (HA) at two different molecular weights (4800 or 17,000 Da, HA 4800 and HA 17000, respectively); (iii) HA-PEG; and (iv) non-decorated liposomes (ND). As a first step, we confirmed the expression of CD44 on THP-1 and RAW 264.7 cells. As previously reported [26], in both undifferentiated and differentiated THP-1 cells, CD44 was highly expressed, with mean fluorescent intensity (MFI) values of 61 and 74, respectively. Moreover, in RAW 264.7 cells, CD44 was expressed to a greater extent than in THP-1 cells, with an MFI value of 137 (Table 2).

In order to verify the hypothesis that phagocytic cells are suitable vehicles for drug delivery, we investigated the interaction between liposomes and phagocytic cells using flow cytometry. RAW 264.7 and THP-1 cells differentiated into macrophages with PMA were then incubated with different types of fluorescently-labeled liposomes.

**Figure 5:**
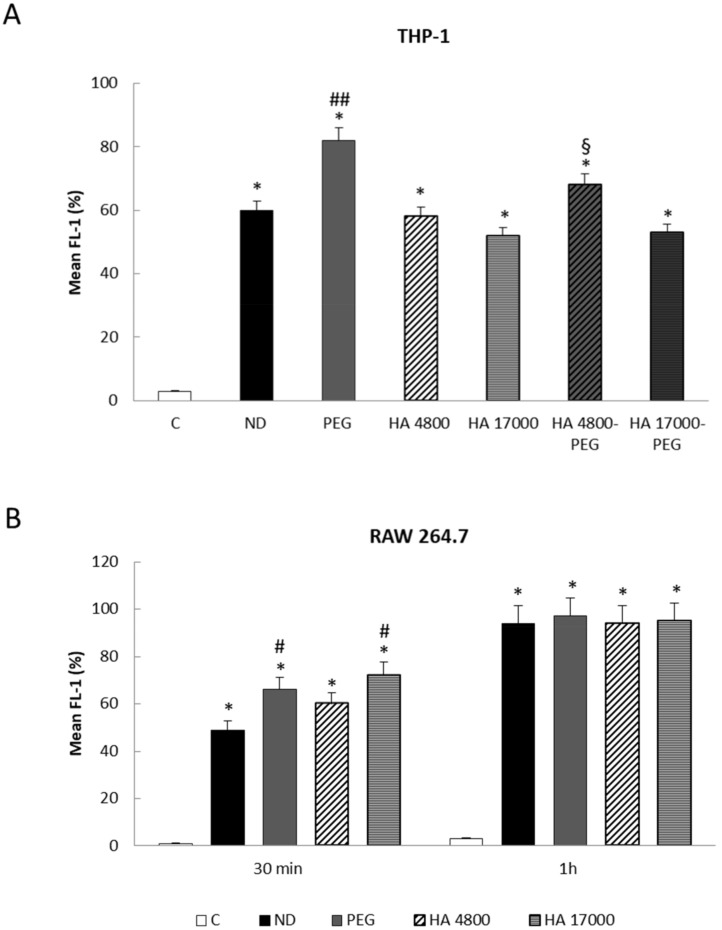
(**A**,**B**) Cellular uptake of different formulations of fluorescein-5-isothiocyanate (FITC) labeled liposomes in THP-1 and RAW 264.7 cells. The internalization of fluorescent liposomes was evaluated in THP-1 (**A**) and RAW 264.7 (**B**) cells by flow cytometry. C = control; ND = non-decorated liposomes; PEG = liposomes decorated with PEG (polyethylene glycol); HA 4800 = liposomes decorated with low molecular weight HA; HA 17000 = liposomes decorated with high molecular weight HA; HA 4800-PEG = liposomes decorated with low molecular weight HA and PEG; HA 17000-PEG = liposomes decorated with high molecular weight HA and PEG. The data in the graphs are expressed as means ± S.D of three independent experiments. * *p* < 0.001 vs. control; ^#^ *p* < 0.01, ^##^ *p* < 0.001 vs. ND liposomes; ^§^ *p* < 0.05 vs. HA 4800 liposomes.

In contrast with some literature [27,28] and with the results obtained in liver cancer cells, we observed a rapid internalization of PEGylated liposomes in THP-1 cells, with a plateau being reached after 1 h of incubation (approximately 85%), compared with the other formulations (Figure 5A). In addition, the presence of PEG seems to improve the cellular uptake efficiency of HA 4800 liposomes (Figure 5A), suggesting that the addition of PEG to liposomes can modify their properties, making them more visible to phagocytic cells. 

A different trend was observed in RAW 264.7 cells in which PEG-coupled liposomes did not show any significant difference in uptake compared with other formulations, as the plateau was reached after 1 h incubation in all cases (Figure 5B).

We have previously demonstrated [26] that the cellular uptake of ND- and HA-coupled liposomes was not reduced by blocking the CD44 receptor with a free high molecular weight (MW) HA (51,000 Da), which suggested that liposome uptake in THP-1 cells occurs via a CD44-independent manner, most probably via the phagocytic activity of macrophages. 

**Figure 6:**
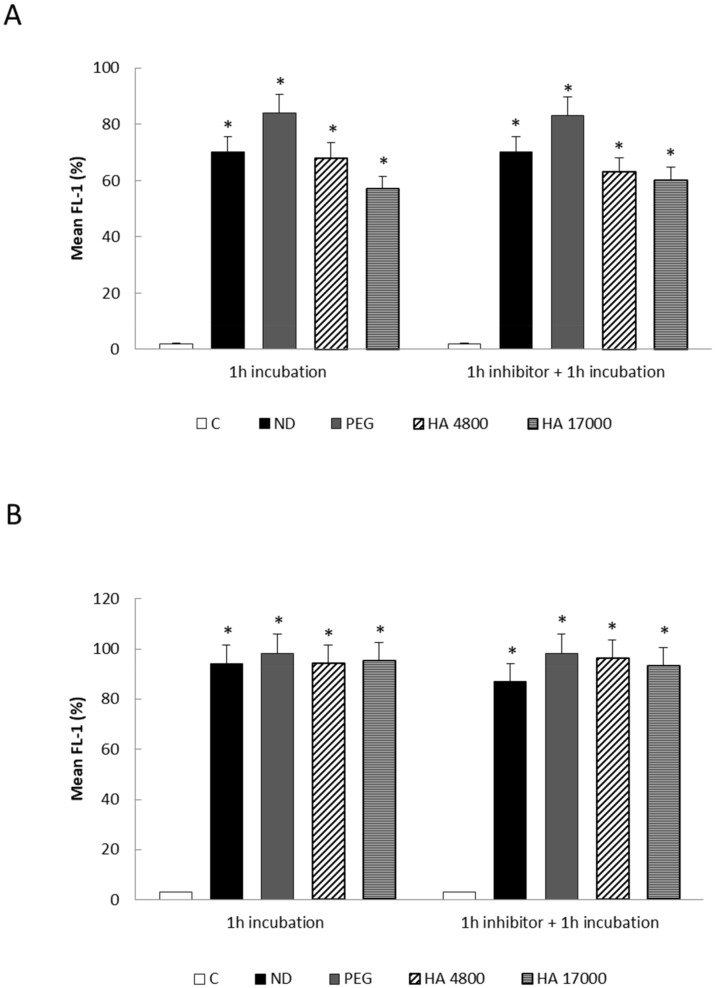
(**A**,**B**) Cellular uptake of different formulations of fluorescein-5-isothiocyanate (FITC) labeled liposomes in THP-1 (**A**) and RAW 264.7 (**B**) cells in the presence of HA inhibitor. The internalization of fluorescent liposomes was evaluated by flow cytometry in THP-1 and RAW 264.7 cells after 1 h pre-incubation with an HA inhibitor (HA 51000). C = control; ND = non-decorated liposomes; PEG = liposomes decorated with PEG (polyethylene glycol); HA 4800 = liposomes decorated with low molecular weight HA; HA 17000 = liposomes decorated with high molecular weight HA. Data in graphs are expressed as means ± S.D of three independent experiments. * *p* < 0.001 vs. control.

This result is also confirmed for PEG-coupled formulations, as the pre-incubation of differentiated THP-1 with a saturating amount of free high MW HA (51,000 Da) did not reduce cellular uptake (Figure 6A). A similar trend was detected in RAW 264.7 cells that were treated with ND- and HA-coupled liposomes (Figure 6B), in which the rapid internalization of the liposomes is probably due to phagocytic activity as well as to the high density of the CD44 receptor on cell surfaces, which can reduce the inhibitory effects of HA 51000. 

### 2.6. THP-1 Polarization

In order to evaluate the biological effect of the liposomes on phagocytic cells, we first investigated the role of our formulations in modulating CD44 expression.

**Figure 7:**
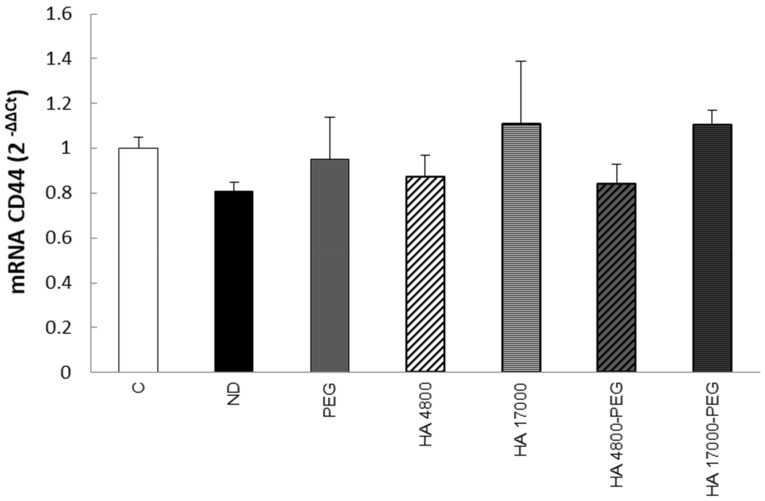
Modulation of CD44 expression by treatment with synthesized liposomes in differentiated THP-1 cells. The quantitative real-time PCR (qPCR) analysis of CD44 expression was conducted in THP-1 cells after 1 h incubation with different liposome formulations. C = control; ND = non-decorated liposomes; PEG = liposomes decorated with PEG (polyethylene glycol); HA 4800 = liposomes decorated with low molecular weight HA; HA 17000 = liposomes decorated with high molecular weight HA; HA 4800-PEG = liposomes decorated with low molecular weight HA and PEG; HA 17000-PEG = liposomes decorated with high molecular weight HA and PEG. The data in the graphs are expressed as means ± S.D of three independent experiments. No significant differences were observed.

As the presence of PEG did not seem to interfere with the cellular uptake of liposomes in RAW264.7 (Figure 6B), we focused our attention on THP-1 cells. As shown in Figure 7, the treatment of differentiated THP-1 cells with the different liposome formulations is unable to increase CD44 transcript levels.

Moreover, considering the relevance of the phenotypic polarization of macrophages in the tumor microenvironment, we next investigated the potential effects of liposome PEGylation in regulating human macrophage polarity.

**Figure 8:**
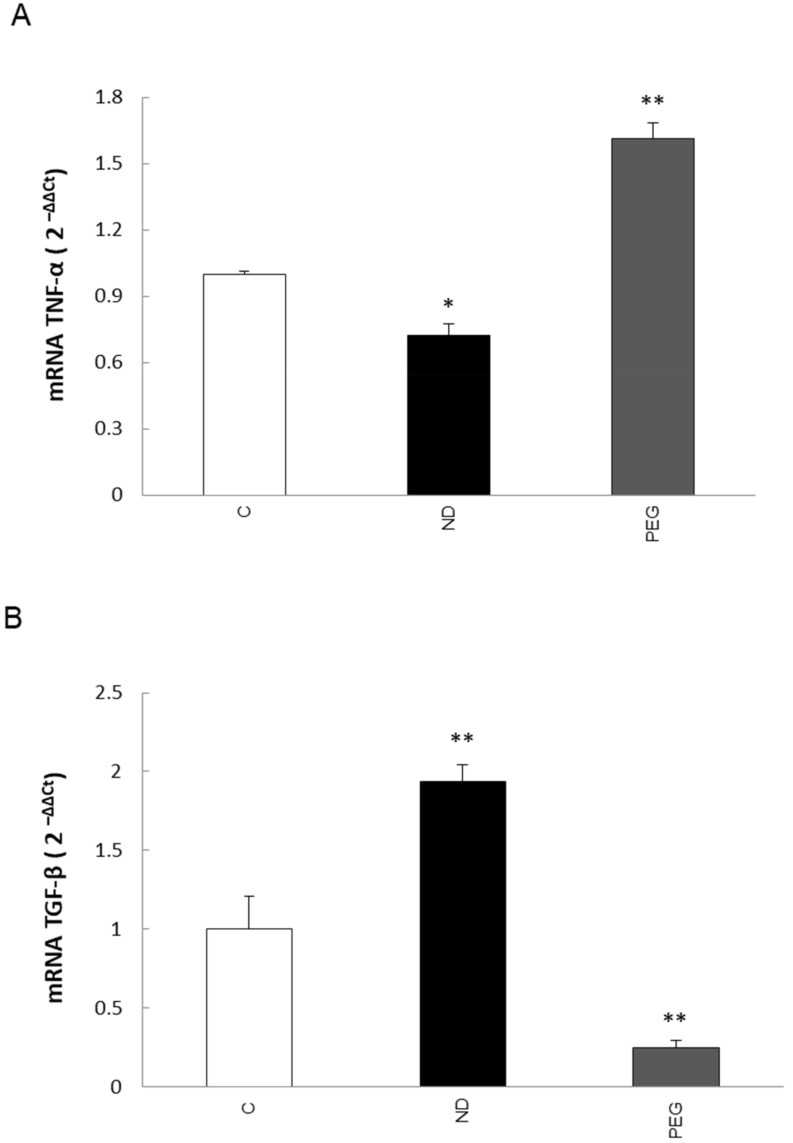
(**A**,**B**) Analysis of transcript levels of TNF-α (**A**) and TGF-β (**B**) in differentiated THP-1 cells. The quantitative real-time PCR (qPCR) of tumor necrosis factor (TNF)-α and transforming growth factor (TGF)-β mRNAs was conducted in THP-1 cells treated for 1 h with ND and PEG liposomes. C = control; ND = non decorated liposomes; PEG = liposomes decorated with PEG (polyethylene glycol). Data in graphs are expressed as means ± SEM of three independent experiments. * *p* < 0.05, ** *p* < 0.001 vs. control.

By performing qPCR analyses, we evaluated the expression of pro-inflammatory cytokine tumor necrosis factor-α (TNF-α) and transforming growth factor–β1 (TGF-β1), representative markers that can identify the M1 or M2 phenotypes, respectively. Preliminary data indicate that differentiated THP-1 cells exposed to 5% PEG-modified liposomes express higher levels of TNF-α transcripts than those exposed to ND liposomes (Figure 8A). In addition, we observed a significant down-regulation in the expression of TGFβ (Figure 8B), thus suggesting that PEG plays a role in the modulation of THP-1 cell polarization towards the M1 phenotype.

## 3. Discussion

HCC is the most frequent primary liver cancer and is ranked as the fifth most common cancer and the fourth leading cause of cancer mortality worldwide, with a minority of patients surviving 5 years from diagnosis, despite treatment. Given the rising levels of obesity and its metabolic complications, HCC incidence is expected to increase in the near future [2]. Moreover, literature data and clinical practice have recently underlined the alarming increase in HCC incidence at earlier stages in non-cirrhotic NAFLD/NASH patients. Liver transplantation, radiofrequency ablation, chemoembolization–TACE, and Sorafenib are the therapeutic strategies that have been indicated by the most recent guidelines for the clinical management of HCC, regardless of its etiology [29]. However, clinical practice for this disease is in need of an innovation in therapeutic interventions in view of the multi-stage carcinogenesis and the many factors (including inflammation, reactive-oxygen-species generation, mitochondrial dysfunction and genetic alterations) and actors (tumor-associated macrophages, TAMs, cancer associated fibroblasts, CAFs, and the tumor microenvironment (TME)) that are involved in and/or influence HCC initiation and progression. In this connection, several strategies to improve clinical benefits and therapeutic outcomes have been introduced, and these include techniques based on peptide-decorated liposomes that target specific receptors for drug delivery [12]. The use of liposomes has vastly improved the therapeutic index of several drugs. In this study, we propose dual-targeted therapy in order to increase treatment efficiency (i.e., better targeting and minimizing the adverse toxic effects on the surrounding organs) by acting on liver cancer cells as well as inflammatory cells. We have introduced dual-functional, HA-modified and/or PEG-modified, liposomes that display different uptake levels in liver cancer cells (HepG2 and Huh7) and inflammatory cells (human and murine macrophages cell lines, THP1 and RAW 264.7, respectively).

The transmembrane glycoprotein receptor CD44 is the major hyaluronan (HA) receptor, and the binding of CD44 to HA has been reported to modulate several phenotypic behaviors in cancer cells, including tumor progression, metastasis, and proliferation. Our data show increased levels of CD44 transcripts in HCC patients of different etiology, compared with cirrhotic patients that did not develop HCC. Moreover, upon analyzing CD44 expression in an experimental murine model of liver carcinogenesis (DEN/CDAA protocol), we observed that CD44 transcripts are increased in mice with NASH (induced using a choline-deficient L-amino acid-defined diet, CDAA), and are further up-regulated in experimental HCC that arise in a NASH background, suggesting that there is a progressive increase in CD44 transcript levels during disease progression. These results are consistent with a meta-analysis reported by Luo and Tan [23], in which the authors underlined an association between CD44 expression and higher tumor TNM stage, as well as poor overall survival (OS) for patients with HCC, which strongly suggests that CD44 plays a role in contributing to HCC development and progression, making it a potential prognostic factor. This correlation between CD44 expression and the aggressiveness of HCC tumors has been confirmed, in this work, in in vitro experiments performed on HepG2 and Huh7 cells (hepatoblastoma and hepatocarcinoma cell lines, respectively). In fact, the analysis of CD44 transcript levels in these two liver cancer cell lines showed an aberrant expression of CD44 in Huh7, in comparison with HepG2 cells in which the CD44 receptor was almost absent, suggesting that CD44 expression progressively increases during the acquisition of a more aggressive phenotype. Our data are consistent with the following literature data: (i) Siracusano and colleagues [22] reported a crucial role for CD44 in maintaining the cancer stemness of Huh7; (ii) CD44 expression was observed to increase in HCC progenitor cells (HcPCs) during cancer progression [30]; (iii) CD44 is necessary for the EMT process in HCC cell lines and is associated with the mesenchymal phenotype [31].

Along these lines, the goal of this study to assess the role of CD44 as a target receptor for drug distribution and the efficacy of HA-modified liposome uptake in liver cancer cells may have some clinical relevance. In fact, we provided evidence for an improvement in HA-modified liposome uptake in cancer cell lines. However, in vitro experiments on HepG2 cells (hepatoblastoma-derived cell line with very low levels of CD44) showed that the uptake of non-decorated liposomes (ND) and HA-modified liposomes was similar, without significant differences, suggesting that liposome internalization was either due to diffusion through the plasma membrane or endocytic events. By contrast, Huh7 cells (hepatocarcinoma cell line, with high levels of CD44 receptor) exhibited an early and prolonged increase in HA-modified liposome uptake, compared with ND liposomes, demonstrating that liposome internalization occurs through a CD44-mediated process. These results confirm CD44 as a putative therapeutic target for improving drug delivery to liver cancer cells.

In consideration of the relevance of the inflammatory tumor microenvironment in HCC progression, the second goal of this study was to evaluate the ability of a mixed formulation of liposomes to differentially target cancer cells and macrophages. Our results suggest that our formulation is a possible solution for the active targeting of liver cancer cells and infiltrating tumor macrophages. In fact, when evaluating liposomes that were conjugated with either HA or PEG (5%) with human and murine macrophage cell lines, we observed a significant difference between the uptake of HA-modified liposomes and PEGylated liposomes, with there being a rapid increase in the internalization of PEG-coated liposomes in THP-1 and RAW264.7. Although our data are in conflict with literature data reporting that PEG chains reduce the internalization of liposomes by the mononuclear phagocytic system [27,28], they seem to be potentially relevant. In fact, limited PEGylated-liposome internalization was observed in HepG2 cells (Supplementary Figure S1, which is why it was not further investigated in cancer cells), meaning that the selective targeting of multiple cellular targets for drug delivery is a real possibility. In addition, this difference in behavior is promising for further drug-delivery applications as macrophages may be able to transport the liposomes in tissues via their ability to migrate and infiltrate the tumor microenvironment. Although literature data have established immune modulatory effects of PEG, its use as a drug-delivery system for cancer treatment is controversial [32]. In this regard, it has been reported that low-PEG-coating-density nanoparticles are preferably accumulated in tumor mass, rather than in the parenchyma, thus exerting an anti-cancer effect [33]. On the other hand, nanoparticles with high-level PEG coatings (over 5%) have longer circulation times and a low level of cellular uptake in the tumor cell, which highlights the importance of balancing cellular-uptake efficiency using PEG-coating density to obtain nanoparticles that are optimized for drug delivery [33].

In this connection, our data showed that the internalization of PEGylated liposomes by differentiated THP-1 cells led to the up-regulation of TNF-α transcripts as well as to the downregulation of TGF-β mRNA levels, suggesting that PEG may play a role in the modulation of macrophage polarity toward M1, which is the pro-inflammatory and anti-tumor phenotype. Interestingly, the preliminary results (not shown) indicate that the internalization of 2%PEG-3%HA-coated liposomes does not induce macrophage polarization, pointing out that it may be possible to use liposomes coated with mixed formulations to prevent/avoid the induction of pro-inflammatory and pro-tumor effects.

In conclusion, in view of the complex scenario of HCC, the data reported in this study support our proposal of an alternative drug-delivery strategy that dually and specifically targets liver cancer cells and infiltrating tumor macrophages to counteract two crucial aspects of HCC progression. In fact, one future perspective of this proposal is the use of hyaluronated liposomes to deliver drugs to liver cancer cells, potentially stopping the proliferation of cancer cells or inducing cancer cell death. On the other hand, PEGylated liposomes and liposomes co-decorated with PEG and HA could be used to (i) specifically target inflammatory cells that infiltrate the tumor mass (TAMs) and (ii) enhance the M1 pro-inflammatory response against tumor cells.

## 4. Materials and Methods

### 4.1. Materials

Sodium hyaluronate, of molecular weight (MW) 4800 (HA 4800) and 17000 (HA 17000) Da, was purchased from Lifecore Biomedical (Chaska, MN, USA). All phospholipids were obtained from Avanti Polar-Lipids, as distributed by Merck Life Science S.r.l. (Milan, Italy). Cholesterol and all the other chemicals were obtained from Merck Life Science S.r.l. Fluorescein-5-(and-6)-sulfonic acid trisodium salt was purchased from Invitrogen, Life Technologies (Monza, Italy). Conjugates between hyaluronic acid (HA) and 1,2- dipalmitoyl-sn-glycero-3-phosphoethanolamine (DPPE) (HA-DPPE) were prepared using the method described in Arpicco et al. [25].

### 4.2. Preparation of Liposomes

Liposomes were prepared using the thin lipid film hydration and extrusion method. The liposomes were composed of 1,2-distearoyl-sn-glycero-3-phosphocoline (DSPC), cholesterol (CHOL), and 1,2-distearoyl-sn-glycero-3-phosphoethanolamine-*N*-[amino(polyethylene glycol)-2000] (mPEG2000-DSPE, indicated as PEG in the text) (55:40:2 molar ratio) or only DSPC/CHOL (55:40 molar ratio, indicated as ND in the text).

Lipids (DSPC 4.35 mg, CHOL 1.55 mg, and either PEG 1.4 mg or PE 0.38 mg) were dissolved in 400 µL of chloroform and evaporated in a rotary evaporator (BUCHI Italia, Cornaredo, Italy). The resulting lipid films were hydrated in 900 µL of HEPES [4-(2-hydroxyethyl) piperazine-1-ethanesulforic acid] buffer (pH 7.4) at room temperature, and the suspension was vortex mixed for 10 min and bath sonicated. The formulations were extruded (Extruder, Lipex, Vancouver, BC, Canada) and the suspension was passed 10 times through a 220 nm polycarbonate membrane under nitrogen (Costar, Corning Incorporated, Corning, NY, USA) at a set temperature of 5 °C above the phase-transition temperature of the lipid mixture. The same method was used to prepare the HA-decorated liposomes [HA 4800, HA 17000 and HA-PEG]: lipid films were either made up of DSPC/CHOL/mPEG2000-DSPE (55:40:2 molar ratio, DSPC 4.35 mg, CHOL 1.55 mg, and PEG 0.6 mg) or DSPC/CHOL (55:40 molar ratio, DSPC 4.35 mg, and CHOL 1.55 mg) and were then hydrated using solutions of the different HA-DPPE conjugates (3 or 5 molar ratios, HA 4800 1.65 mg or 2.75 mg, HA 17000 5.31 mg or 8.85 mg). Fluorescent-labeled liposomes were prepared as described above, and a 10 mM solution of fluorescein-5-(and-6)-sulfonic acid trisodium salt in HEPES buffer was used during hydration. Liposomes were purified of non-entrapped fluorescein-5-(and-6)-sulfonic acid trisodium salt using chromatography on Sepharose CL-4B columns, eluting with HEPES buffer. Liposomes were stored at 4 °C.

### 4.3. Liposome Characterization

Liposomes were characterized in terms of mean particle size, the polydispersity index (PI), and surface charge. The parameters were determined at 25 °C using quasi-elastic light scattering (QELS) in a nanosizer (Nanosizer Nano Z, Malvern Inst., Malvern, UK). The selected angle was 173° and the measurement was performed after the nanoparticle suspension was diluted in MilliQ^®^ water. Each measure was performed in triplicate. Moreover, the liposome surface charge was determined using zeta potential measurements at 25 °C in the Nanosizer Nano Z and the Smoluchowski equation. Measurements were carried out in triplicate.

### 4.4. Cell and Culture Conditions

The human monocytic leukemia cell line (THP-1 cells), murine macrophages (RAW 264.7 cells), the hepatoblastoma cell line (HepG2 cells), and hepatocarcinoma cells (Huh7) were obtained from American Type Culture Collection (ATCC, USA) and maintained in RPMI (THP-1) and DMEM media (RAW 264.7, HepG2 and Huh7) (Sigma Aldrich Spa, Milan, Italy) supplemented with 10% fetal-bovine serum, 100 U/mL penicillin, 100 μg/mL streptomycin, and 25 μg/mL amphotericin-B (Sigma Aldrich Spa, Milan, Italy). For all experiments, THP-1 cells were seeded 7 × 10^5^ in 35 mm petri dishes, differentiated for 48 h with phorbol 12-myristate 13-acetate (PMA, 50 nM), and cultured for 24 h in fresh medium. RAW 264.7 were seeded 5 × 10^5^ in six-well culture plates and cultured for 24 h. The cells were then treated with the different liposome formulations (ND, 5%PEG, 5%HA 4800, 5%HA 17000, 3%HA 4800-2%PEG, 3%HA 17000-2%PEG). For the liver cells, HepG2 and Huh7 cells were seeded 5 × 10^5^ in 35 mm petri dishes and then treated with different liposome formulations (ND, HA 4800, HA 17000).

### 4.5. Cellular Uptake

A quantitative determination of the cellular uptake was performed using flow cytometry. Differentiated THP-1 cells were seeded in six-well culture plates and exposed, for different times, to FITC-labeled ND, PEG, HA 4800, HA 17000, HA 4800-PEG, and HA 17000-PEG liposomes. After the medium was removed, the cells were washed twice with PBS, collected using a cell scraper, and finally re-suspended in 1 mL of PBS. RAW 264.7 cells were seeded in six-well culture plates and exposed, for different times, to FITC labeled liposomes. After the medium was removed, the cells were washed twice with PBS, collected by trypsinization, and finally re-suspended in 1 mL of PBS. Cells were also pre-incubated with a 100× molar excess of free high molecular weight HA (MW 51,000 Da, indicated as HA 51000 in the text) for 1 h. For the liver cancer cells, HepG2 and Huh7 cells were seeded 5 × 10^5^ in six-well dishes and then treated with different liposome formulations (ND, HA 4800, HA 17000). For all experimental conditions, the intracellular uptake of the FITC-labeled modified liposomes was analyzed using a FACSCanto cytometer (Becton Dickinson, Franklin Lakes, NJ, USA). Cells incubated in the absence of the liposomes were used as controls. Approximately 5000 gated events were acquired for each sample. Flow cytometry data were processed, analyzed, and graphed using CellQuest software (Becton-Dickinson).

### 4.6. Analysis of CD44 Protein Expression

The cell surface expression of CD44 was detected by staining cells with the fluorochrome-conjugated CD44 antibody in the dark at room temperature for 15 min. The isotype-matched irrelevant antibody was used to set background signals. Cells were washed with PBS 1X solution and then analyzed on a FACSCanto cytometer (Becton Dickinson). Approximately 10,000 gated events were acquired for each sample. Flow cytometry data were processed, analyzed, and graphed using CellQuest software (Becton-Dickinson).

### 4.7. Quantitative Real-Time PCR (Q-PCR)

RNA extraction, complementary DNA synthesis, and quantitative real-time PCR (Q-PCR) reactions were performed as previously described [34]. Human TNF-α, TGF-β, and CD44 mRNA levels from THP-1 cells as well as CD44 transcript levels from HepG2 and Huh7 cells were measured by Q-PCR, using the SYBR^®^ green method as previously described [34]. The amplification mix was prepared using the Roche LightCycler FastStart DNA MasterPLUS SYBR Green I kit according to the manufacturer’s instructions, and real-time PCR was performed using a LightCycler instrument. The oligonucleotide sequences of primers used for RT-PCR are reported in Table 3. Gliceraldehyde-3-phosphate dehydrogenase (GAPDH) was used as an internal reference and co-amplified with target samples using identical Q-PCR conditions. Samples were run in triplicate and mRNA expression was generated for each sample. The specificity of the amplified PCR products was determined via melting curve analysis and confirmed using agarose gel electrophoresis.

### 4.8. Human Samples

In this study, we analyzed different patient cohorts: (i) a selected cohort of cirrhotic patients of different etiology (*n* = 10) who were referred to the Division of Gastro-Hepatology at the University of Turin; (ii) liver specimens from HCC patients (*n* = 67), with similar mixed etiology (alcohol, HCV, HBV, autoimmune, metabolic), who were referred to the Internal Medicine and Hepatology Clinic, Department of Medicine, University of Padova. Tissue samples were collected at the time of liver tumor resection or liver transplantation. All subjects gave informed consent to the analysis, and the study protocols conformed to the ethical guidelines of the 1975 Declaration of Helsinki and were approved by the ethics committees of the Azienda Ospedaliera Universitaria Città della Salute, Torino, Italy (for cirrhotic patients, n. 0125391, on 18 December 2018, Practice n. CS2/880), and of the Azienda Ospedaliera-Università di Padova (on 11 December 2006, for HCC patients).

### 4.9. Animal Experiments

In this study, wild type C57BL/6 mice were submitted to the following: (a) an established liver carcinogenic protocol involving a single administration of diethylnitrosamine (DEN, 25 mg/kg bw, i.p.) at the age of 2 weeks to mice that were fed, from the age of 6 weeks, on a choline-deficient L-amino acid-defined (CDAA) diet (Laboratorio Dottori Piccioni, Gessate, Italy) for an additional 26 weeks [35]; (b) a CDAA diet only; or (c) a choline-sufficient L-amino acid-defined (CSSA) diet (Laboratorio Dottori Piccioni, Gessate, Italy) for 24 weeks starting from 6 weeks of age, as previously described [35]. Mice were kept under specific pathogen-free conditions and maintained with free access to pellet food and water. Liver samples were obtained and immediately used/processed for morphological or molecular biology analyses, or frozen and thereafter maintained at −80 °C for further analysis. These experiments complied with EU and national ethical guidelines for animal experimentation, and the experimental protocols were approved by the Animal Ethics Committee of the University of Eastern Piedmont, Novara, Italy, and the Italian Ministry of Health (authorization n°1114/2016).

### 4.10. Statistical Analysis

For human samples and animal experimental models, we performed statistical analyses using GraphPad Prism 6.01 statistical software (GraphPad Software, San Diego, CA, USA). In particular, for human analyses and animal experimental models, the Mann–Whitney non-parametric test and the one-way ANOVA test with Tukey’s correction for multiple comparisons were employed to evaluate the statistical significance of the data, with *p* < 0.05 being considered significant. The Kolmogorov–Smirnov test was used to preliminarily assess the normality distribution. Statistical differences in in vitro experiments, between control cells and cells treated with different formulations of liposomes, were evaluated using the Student’s *t*-test or one-way ANOVA analysis. A value *p* < 0.05 was considered statistically significant. The in vitro data presented in bar graphs represent means ± SD and were obtained from average measures of at least three independent experiments.

## Figures and Tables

**Table 1:** Characteristics of plain and HA liposomes.

Formulation	Size (nm)	PDI	Zeta Potential
ND	171 ± 2	0.113	−16 ± 1.2
PEG	165 ± 3	0.116	−14 ± 0.9
HA 4800	198 ± 1	0.173	−29 ± 1.1
HA 17000	225 ± 2	0.175	−40 ± 1.8
HA 4800-PEG	180 ± 2	0.117	−22 ± 1.3
HA 17000-PEG	210 ± 1	0.109	−24 ± 1.6

Values are the means ± SEM of three independent experiments, each performed in triplicate.

**Table 2:** Mean fluorescence intensity (MFI) of CD44 expression as analyzed by flow cytometry.

Cell Line	MFI
THP-1 monocytes	61.11
THP-1 macrophages	74.46
RAW 264.7 macrophages	137

**Table 3:** Oligonucleotide sequences of primers used for RT-PCR.

Primer	Sense	Reverse
Human *TNF-α*	5′-AACCTCCTCTCTGCCATCAA -3′	5′-GGAAGACCCCTCCCAGATAC-3′
Human *TGF-β*	5′-GGGACTATCCACCTGCAAGA-3′	5′-CCTCCTTGGCGTAGTAGTCG-3′
Human *GAPDH*	5′-TGGTATCGTGGAAGGACTCATGAC-3′	5′-ATGCCAGTGAGCTTCCCGTTCAGC-3′
Human *CD44*	5′-ACACACGAAGGAAAGCAGGA-3′	5′-CACTGGGGTGGAATGTGTCT-3′

## Data Availability

Not applicable.

## Supplementary Materials

The following supporting information can be downloaded. Figure S1: Cellular uptake of PEG-coated FITC-labeled liposomes in liver cancer cells.
