# Supplementary material for: Hyaluronated and PEGylated Liposomes as a Potential Drug-Delivery Strategy to Specifically Target Liver Cancer and Inflammatory Cells
Source: Molecules. 2022 Feb 4;27(3):1062. doi: 10.3390/molecules27031062 (PMC8840578; doi:10.3390/molecules27031062)
Supplement: Supplementary file 1 [file molecules-27-01062-s001.zip › molecules-1554320-SI.pdf]

Figure S1

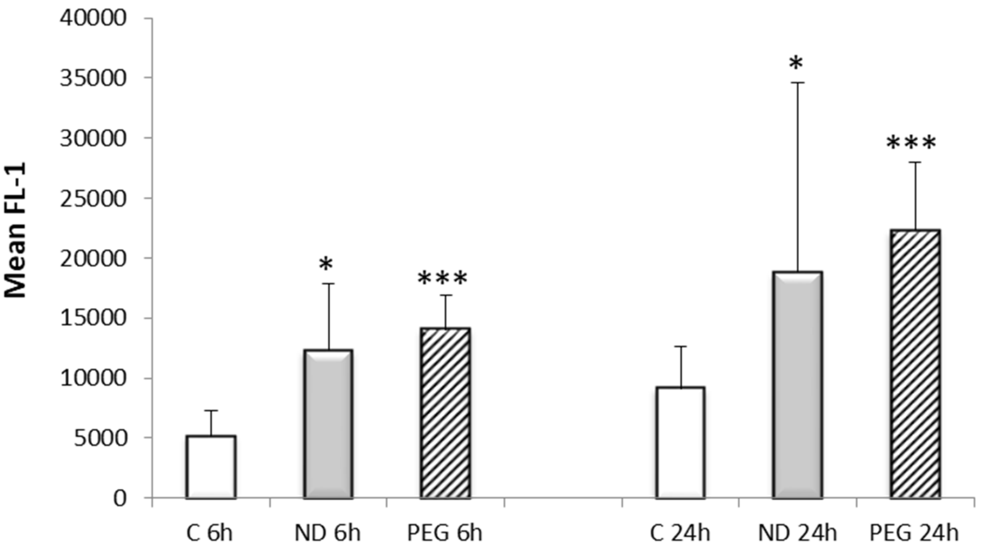

Figure S1. Cellular uptake of PEG-coated FITC labelled liposomes in liver cancer cells. Flow cytometry analysis of internalization of fluorescent liposomes in HepG2 and Huh7 after incubation of cells with synthetized PEG-coated liposomes at the indicated time points. Data in graphs are expressed as means ± S.D of three independent experiments (*$p<0.05$, ***$p<0.001$ vs control; no significant differences was observed vs ND liposomes).
